# Privacy and Trust in eHealth: A Fuzzy Linguistic Solution for Calculating the Merit of Service

**DOI:** 10.3390/jpm12050657

**Published:** 2022-04-19

**Authors:** Pekka Ruotsalainen, Bernd Blobel, Seppo Pohjolainen

**Affiliations:** 1 Faculty of Information Technology and Communication Sciences (ITC), Tampere University, 33100 Tampere, Finland; seppo.pohjolainen@tuni.fi; 2 Medical Faculty, University of Regensburg, 93953 Regensburg, Germany; bernd.blobel@klinik.uni-regensburg.de

**Keywords:** privacy, trust, modelling, antecedents, Fuzzy attractiveness rating

## Abstract

The use of eHealth and healthcare services are becoming increasingly common across networks and ecosystems. Identifying the quality and health impact of these services is a big problem that in many cases it is difficult determine. Health ecosystems are seldom designed with privacy and trust in mind, and the service user has almost no way of knowing how much trust to place in the service provider and other stakeholders using his or her personal health information (PHI). In addition, the service user cannot rely on privacy laws, and the ecosystem is not a trustworthy system. This demonstrates that, in real life, the user does not have significant privacy. Therefore, before starting to use eHealth services and subsequently disclosing personal health information (PHI), the user would benefit from tools to measure the level of privacy and trust the ecosystem can offer. For this purpose, the authors developed a solution that enables the service user to calculate a Merit of Service (Fuzzy attractiveness rating (FAR)) for the service provider and for the network where PHI is processed. A conceptual model for an eHealth ecosystem was developed. With the help of heuristic methods and system and literature analysis, a novel proposal to identify trust and privacy attributes focused on eHealth was developed. The FAR value is a combination of the service network’s privacy and trust features, and the expected health impact of the service. The computational Fuzzy linguistic method was used to calculate the FAR. For user friendliness, the Fuzzy value of Merit was transformed into a linguistic Fuzzy label. Finally, an illustrative example of FAR calculation is presented.

## 1. Introduction

Nowadays, people use digital services such as e-commerce, online shopping and, increasingly, eHealth services, nearly every day. These services are often built on platforms that—together with different stakeholders—form an ecosystem, where transactions take place without physical contact [1,2]. Although information privacy, security and trust are major concerns in digital markets, researchers have observed that digital information systems are seldom designed with privacy in mind [2]. Tan found that digital information systems are unreliable, unsecure and risky, and service providers deploying them have the power, tools and intention to manipulate their users’ (a person or patient) trusting beliefs [3]. The assumption that a user can control the use of their personal information on the Internet and in ecosystems is only an illusion. In fact, we simply do not have privacy [4,5,6]. In real life, it is nearly impossible for the service user (SerU) to prevent unnecessary data collection, and to know to whom data is disclosed [7]. Often, the SerU is unaware and lacks understanding of actual privacy threats and their possible consequences [8]. Unfortunately, she/he cannot expect that domain-specific laws guarantee privacy and trust [9]. Instead, personal information is often disclosed and distributed to other stakeholders across health ecosystems without the user’s consent or an awareness of privacy policies [10]. Frequently, the only choice for a SerU is to blindly trust the service provider (SerP) or to reject the service [11].

Today’s eHealth services and applications offer many health promises, but to be beneficial they require a large amount of personal health information (PHI), such as vital signs, lifestyle, psychological characteristics and personal health beliefs. The SerU’s education and socioeconomic status are also often exploited [12]. The ongoing transition towards personalized, participative, preventive, predictive and precision health and social care requires even more PHI, such as personal behaviors, social relations and environmental data [12]. A significant privacy concern is that PHI is not collected and used just by regulated healthcare organizations, but also by commercial web service providers and social web applications. PHI is also shared across eHealth ecosystems between stakeholders following different business models. These facts raise meaningful privacy and trust concerns. They result from the insufficiency of security-oriented privacy protection tools currently used in eHealth, such as access control, consent, and data anonymization. Furthermore, data encryption has limited power, as eHealth applications frequently need PHI in plain form [13].

From a privacy and trust point of view, the current situation is unsatisfactory. To enjoy the health benefits offered by eHealth, personal health apps and precise health services, the SerU has to maintain information privacy and know how much trust to place in a service provider and in the ecosystem, and what the level of actual offered privacy is. To meet this challenge, the authors have developed a solution that enables the SerU to calculate the level of trust and privacy to place in online eHealth ecosystems.

## 2. Definitions

Many of the terms used in this research do not have clear meaning. In this paper, the following definitions are used:

- Attitude is an opinion based on beliefs. It represents our feelings about something and the way a person expresses beliefs and values [10];
- Belief is the mental acceptance that something exists or is true without proof. Beliefs can be rational, irrational or dogmatic [14];
- eHealth is the transfer and exchange of health information between health service consumers (subject of care), health professionals, researchers and stakeholders using information and communication networks, and the delivery of digital health services [15];
- Harm is a potential direct or indirect damage, injury or negative impact of a real or potential economic, physical or social (e.g., reputational) action [11];
- Perception refers to the way a person notices something using his or her senses, or the way a person interprets, understands or thinks about something. It is a subjective process that influences how we process, remember, interpret, understand and act on reality [16]. Perception occurs in the mind and, therefore, perceptions of different people can vary;
- Reputation is a related but distinct concept of trust. It can be considered as a collective measure (a common opinion or recommendation) of a community about a trustee [17,18];
- Risk is a subjective expectation of loss, and the probability, likelihood or possibility of something that people fear as negative [19]. Consequently, risk perception is a feeling, impression, judgement and subjective evaluation about the likelihood of negative occurrences [20].

## 3. Methods

**Figure 1:**
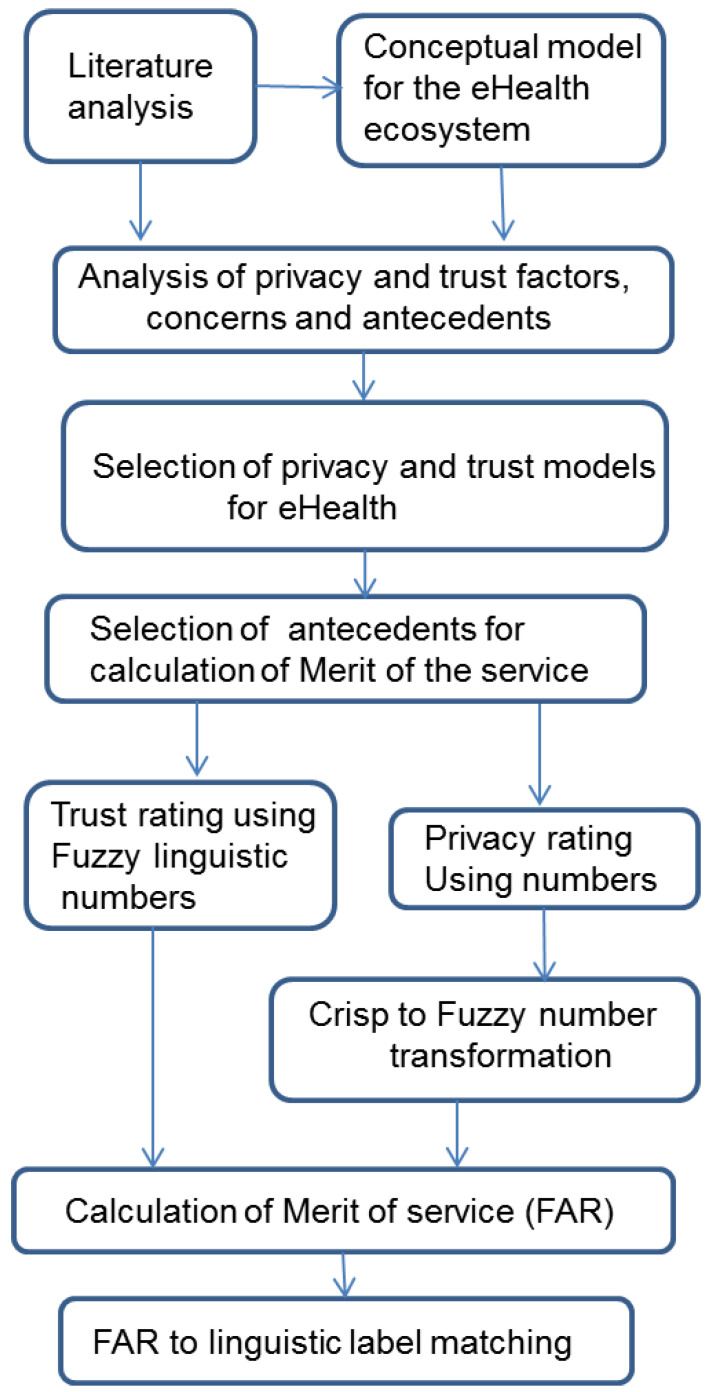
Phases of the study.

This study drew from existing privacy, trust, e-commerce, Internet shopping, and eHealth literature. Instead of defining separate privacy and trust scores, a Merit of Service as a combination of privacy, trust and expected health impact was calculated for the service used as a whole. Figure 1 shows the different phases of this study. Methods such as literature analysis, system analysis, modelling, Fuzzy mathematics and heuristics were used.

In this study, the first step was a deep literature analysis followed by the development of a conceptual model for the eHealth ecosystem. Up to 480 research articles covering different views on e-commerce, Internet shopping, privacy, trust and eHealth published in major journals were reviewed in detail. Because e-commerce, Internet shopping and eHealth build on the same format of ICT architecture and technology, concerns researchers have found in e-commerce and Internet shopping were also expected to exist in eHealth services that are modelled ecosystems. Appropriate privacy and trust models for eHealth, and privacy and trust attributes for calculating the Merit of Service, were selected using a heuristic method and findings were obtained from the literature analysis.

The Fuzzy attractiveness rating (FAR) method was used for calculating the Merit of eHealth service. The value of Merit was calculated using a linguistic Fuzzy approximation method, where the input attributes were Fuzzy trust rating, linguistic privacy value, and expected quality of service. Personal weights for attributes were also supported. To make the result (a linguistic FAR number) user-friendly, it was finally transformed into a Fuzzy linguistic label.

## 4. Related Research

Privacy is an elusive concept that has been studied as a philosophical, psychological, sociological, behavioral, economical and legal concept [19,21]. Traditionally, privacy is understood as an interpersonal concept, but today we understand that it exists in person–computer, computer–computer, and person–organization contexts. Two basic modes of privacy are general privacy and contextual privacy. Basic approaches for general privacy are value-based (e.g., human rights) or cognate-based, where privacy is related to the individual’s mind, perception and cognition [19,22]. Privacy violations involve harm to individuals that can also take place in the future [21].

Widely used privacy approaches include privacy as an individual’s right to control; privacy as a commodity, property, contextual integrity, a behavioral concept and social good; privacy as a concern or legal construct; risk-based privacy; and privacy as a fiducial duty [23,24]. The focus of control theory is self-determination regarding personal information. Modern control approaches see privacy as the ability to control access to the self [22,23]. In Pertronio’s boundary theory, people control information flow through boundaries [23]. According to Lilien, privacy is “the right of an entity acting on its own behalf, to determine the degree to which it will interact with its environment, including the degree to which the entity is willing to share information about itself with others” [25]. 

The concept of privacy as a commodity understands privacy as economic good that can be traded for other goods or services [22,26]. In the model of privacy as personal property, the person has data ownership [27,28]. Privacy as a concern refers to individuals’ anxiety regarding data collectors’ and processors’ information practices [20]. Privacy as a regulative (legal) construct tries to regulate the disclosure and use of information in a context, and to protect individuals [27]. The risk-based approach to privacy focuses on risk (e.g., social discrimination, negative impacts of data misuse, surveillance and behavioral manipulation) caused by data collection, use and disclosure [19]. Risk includes uncertainty, and in real life it is difficult or impossible for the SerU to measure the actual level of privacy risk at play [19].

Consumer privacy and online privacy are contextual privacy approaches used in consumer-to-business relationships (e.g., in e-commerce and Internet shopping) [29]. Online privacy can be understood as the level of privacy a user has on the Internet and social networks.

The vague and context-dependent nature of privacy and the lack of reliable information available make the measurement of actual (objective) privacy challenging [30]. Therefore, different proxies such as disposition, belief, expectation, perception, service-level agreements, contracts, external third-party seals, service provider’s privacy policy documents, reputation, audit trails, direct observations, and degree of compliance with standards and risk are widely used [25,31,32]. Unfortunately, all of these have weaknesses. Belief is a personal trait, disposition is a psychological prerequisite, and neither can be measured [33]. In real life, a SerU has almost no chance to negotiate a service-level agreement (SLA) or to make a contract with the service provider. Third-party seals and certification are seldom available for the eHealth user, and the current security-oriented access-control solutions are ineffective. Privacy damage frequently takes place after the incident, and risks and perceptions are often only opinions [13].

Researchers have developed many methods for calculating or estimating levels of privacy, such as privacy calculus, risk evaluation and assessments, privacy threat analysis, regulatory compliance analysis, the evaluation of privacy documents and privacy policy compliance, and the privacy level of an information system [25]. In these calculations, the SerP’s privacy features and user’s privacy concerns are typically used. Regarding the privacy calculus method, it assumed that individuals can rationally estimate and weigh risks, and maximize benefits. According to Kruthoff, the assumption that people are aware of the risk is seldom true; therefore, the privacy calculus is not a good solution [34]. According to Mitchell, objective risk is a good proxy for privacy but, unfortunately, it cannot be measured in real life [35]. 

Similarly to privacy, trust is a vague concept that is defined in various ways in different cultures and contexts [9]. Trust exists in the relationship between a trustor and trustee, and it is widely understood as a subjective feature, psychological state, and personal trait, and it is the prerequisite of an action [9,36,37]. Trust has been studied from the viewpoints of philosophy, psychology, social sciences, information science, and economy. Basic trust types are general trust that has no relation to features of the trustee, and domain-specific trust [38]. Interpersonal trust takes place between humans, but the trustor/trustee can be any entity, such as an organization, institution or artefact [39]. Typically, trust is needed in situations where the trustor has insufficient information about the features and behaviors of the trustee [39]. Disposition (propensity) to trust is the tendency to trust others [40]. Trust is also widely understood as a belief, expectancy or feeling [41]. According to Castelfranchi, trust is, at the same time, a mental attitude towards another agent and a simple disposition to rely upon the other [42]. Chen defined trust as an intention to accept vulnerability under the conditions of risk caused by a trustee’s actions [36]. A widely used definition of trust is “The willingness of a party to be vulnerable to the actions of another party based on the expectation that the other will perform a particular action important to the trustor, irrespective of the ability to monitor or control that other party.” [38,41,43]. For Gambetta, “trust is a particular level of the subjective probability with which an agent will perform a particular action, both before one can monitor such action and in a context in which it affects own action” [9]. Economic perceptions of trust are based on calculations, i.e., rational choice mechanisms [38]. Trust and risk are interrelated concepts, i.e., trust is only needed if risk is involved. For Mayer, trust in fiduciary relationships is based on belief in the professional’s competence and integrity [44]. 

Trust (or lack of trust) is one of the major problems in digital environments, e.g., in information systems, computer–computer and human–computer interactions, e-commerce, Internet shopping, social networks, smart physical environments, mobile networks and eHealth [45]. Computational trust imitates the human notion of trust, and it is widely used to substitute mental trust models [18]. It helps the SerU to estimate the degree of trust in a situation. Methods such as intuitive formula, simple mathematics (e.g., mean value, weighted average, weighted rank), probabilistic approaches, cost/benefit calculations, risk evaluations, recommender systems, game theory, utility theory, entropy, belief calculus, subjective logic, collaborative filtering, calculations using linguistic variables, analytic hierarchy processes, use of regression models, and machine learning are widely used for computational trust [18,38,46,47,48,49,50,51,52,53,54]. According to Nefti and Liu, major challenges with computational methods regard how to quantify trust, the lack of sufficient and reliable (direct) information, and uncertainty in attributes [48,55]. 

The Fuzzy nature of trust makes it logical to use Fuzzy logic in presenting and calculating levels of trust. This process has many advantages: Fuzzy logic is a computational method that is capable of using imprecise data and quantifying uncertainty [55]. Furthermore, Fuzzy logic is able to present measurement values and results in linguistic terms, such as “low”, “high” and “good” [56]. 

For modelling and calculation, Fuzzy trust methods such as simple arithmetical operations (e.g., Fuzzy mean, simple additional weighting, and Fuzzy weighted average), Fuzzy distance measurement, Fuzzy multicriteria decision making, the Fuzzy analytic hierarchy process, and Fuzzy attractiveness ratings [57,58,59] are used. Truong et al. developed a reputation and knowledge-based Fuzzy trust service platform for the calculation of personal trust in IoT environments using utility theory [56]. Mahalle et al. used a utility function to calculate the overall trust value for a service using attributes such as experience and knowledge [60]. In a solution developed by Nefti et al., Fuzzy trust was used to evaluate a merchant’s trust in e-commerce. Thereby, attributes such as the existence of a provider, policy fulfilment, and affiliation were deployed [55]. Lin et al. used linguistic terms describing weights of criteria and values of ratings to calculate Fuzzy attractiveness ratings (FARs) for different bids [61].

According to Herrera et al., there are situations where information cannot be presented in crisp numbers. Instead, a qualitative linguistic approach should be used where values of variables are described with words. The value of a variable is characterized by a label (a word), and the meaning is presented as a Fuzzy membership function [62].

Fuzzy logic-based trust solutions have also been used in health care in topics such as medical decision-making, patient monitoring, supporting diagnosis, the analysis of medical (big) data, the quality evaluation of health care services, the analysis of personal health and the creation of Fuzzy healthcare systems [63,64,65,66,67].

## 5. Solution to Calculate the Merit of eHealth Services

### 5.1. Conceptual Model for the eHealth Ecosystem

**Figure 2:**
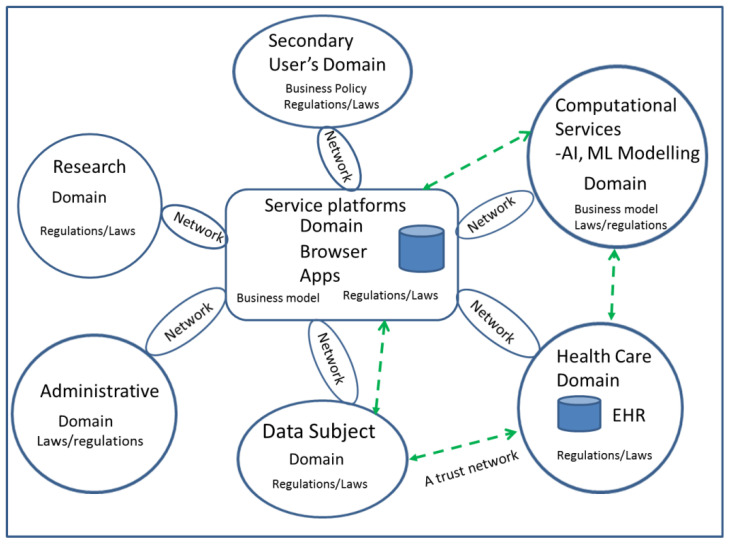
A conceptual model of the eHealth ecosystem.

People use eHealth services and disclose their PHI for applications to obtain personal health benefits. At the same time, they intend to maintain privacy, and to trust in the service provider and in the ICT technology used. Nowadays, eHealth services are increasingly offered via eHealth ecosystems. To understand how this functions and which trust and privacy relations and challenges exist in eHealth ecosystems, a conceptual model has been developed (Figure 2). Typical stakeholders in the eHealth ecosystem are the SerU (a primary source of PHI), health service providers, secondary users of PHI, computational service providers, communication service providers, the service platform operator, and regulators. The platform orchestrates health applications and information sharing in the network. The Internet and mobile networks are typically used for communication. According to Vega et al., typical eHealth websites include portal sites, support groups, charity sites, governmental sites, pharmaceutical sites, sales sites, personal sites, medical databases, media sites and clinical sites [32]. Health services offered by them include health promotion (e.g., nutrition, personal activity), self-care, and self-assessment, forecasting of future disease risk, and different test-, disease- and health-specific information services [68]. The delivery of health services needs large amount of PHI, such as information about conditions, treatments, symptoms, outcomes, laboratory results, genetic information, and health survey responses [6]. An eHealth application collects, processes and stores PHI, and it can also share PHI with other partners in the ecosystem.

### 5.2. Privacy and Trust Challenges in eHealth Ecosystems

In an ecosystem, stakeholders can locate different domains, having their own business models and domain-specific laws/regulations with their own privacy policies and trust features. The main features which separate eHealth ecosystems from e-commerce ecosystems are summarized in Table 1. 

The SerU’s challenge is to find answers to the questions: “How shall I trust the faceless and the intangible?” [39] Who are the stakeholders in the system? Who are the data sharers? Who else can see and use my data? Who has control over my data, and how long it is stored? Furthermore, he or she needs to know the level of trust and privacy of the whole ecosystem, what kind of actual privacy risks exist, and what the harmful future effects of data misuse are. The SerU’s concerns are linked to the lack of reliable and precise privacy and trust information, such as: to which unknown partners and purposes PHI is disclosed; data ownership; whether the SerP and other stakeholders will behave as expected and follow ethical rules and regulatory requirements; whether PHI is sold for direct marketing purposes; and the legitimacy and presence of the vendor, and the quality of the health services offered [58,68,69,70]. Furthermore, it is difficult for the SerU to know which laws and regulations are applied by a certain stakeholder [71]. Often, the SerP’s privacy policy document does not explain which protection tools and procedures required by law are actually implemented [19]. Furthermore, tamper-proof audit trails are seldom available, and even if policy documents are available, they do not explain precisely how PHI is processed [72].

In real life, service providers often expect that the SerU’s privacy needs can be balanced with the providers’ business needs [73,74]. SerU’s and providers can also have contradictorily opinions concerning who “owns” the user’s data. Additionally, often, a service provider assumes the right to use PHI for their own purposes, and share or sell it to business partners [75]. Gomez et al. found that most websites use personal information for customized advertising, and many “trusted” firms share data with their affiliated companies [22]. Furthermore, commercial service providers often have minimal incentives to enforce strong privacy policies [76], and they do not always do what they promise in their policy documents and trust promises. In eHealth, the situation is not much better. Huckvale et al. found poor information privacy practices in health apps [68]. According to Papageorgiou et al., many eHealth service providers failed to provide even basic privacy protection. According to their review, 80% of health apps transmit users’ health-related data, and 50% of apps send data to third parties without encryption [77]. 

### 5.3. Privacy and Trust Models for eHealth

The different privacy and trust approaches discussed in Chapter 4 present different views on privacy and trust, with different factor weights. Therefore, for the calculation of the level of privacy and trust in eHealth ecosystems, it is necessary to choose appropriate models. In this research work, a heuristic method was deployed.

As eHealth services are used in specific contexts, the general privacy approach cannot be successful. Researchers have found (Chapter 4) that a control approach is only an illusion, and from the SerU’s point of view, privacy as commodity, social good, and contextual integrity approaches are insufficient [13]. Because the SerU is unable to utilize information systems and program codes, he or she cannot know the actual privacy risks or estimate the impacts of possible harm. Furthermore, risk perception and probabilities are only subjective opinions and, for the user, it is impossible to know to what extent they represent the actual risks. Therefore, the privacy as risk approach is not suitable for eHealth. According to Kosa, information privacy is about legislation and compliance [78], and because Internet users often have limited knowledge of the SerP’s privacy features and no power to protect their data, they must rely on laws and regulations [79]. Based on the analysis performed above, the authors’ state that, in eHealth ecosystems, a good privacy model is to understand privacy as a personal property [27], and to use legal norm (law) responsibilities and privacy policies as proxy. A benefit to this approach is that both laws and organization’s privacy policy documents are often publicly available, and the privacy as property approach enables the SerU to decide what PHI to disclose and what to protect.

Dispositional trust and trusting belief models are widely used in e-commerce. McKnight has proposed a human disposition to trust technology, as well as trusting beliefs and trusting intentions for information systems [80]. The authors’ state that reducing trust to a personal trait (i.e., propensity to trust) and belief has meaningful weaknesses. Both are strong personal feelings without connection to actual trust in information systems and data processing, and user’s beliefs and feelings are easy to be manipulated by the service provider. Therefore, disposition and trusting beliefs cannot be used in eHealth. The approach comprising willingness to be vulnerable to the actions of another party (Chapter 4) also does not work, because it is based on belief or feelings [81]. Furthermore, trust as subjective probability is not useful, because it is only an opinion, and the definition of realistic probability is frequently impossible. The economistic rational choice model approach also fails because of the limited capacity of humans to make rational choices.

Based on the aforementioned analysis, the authors’ selected a computational trust model. It has many advantages, such as imitating human trust and enabling the service user to compute the level of trust in a context using measurable attributes, such as direct experiences, historical (past) information of the SerP’s features and behaviors, and it also takes into account the SerU’s perceptions [48]. Computational methods are mathematically formulated algorithms which can be quite easily programmed and implemented. As the computational linguistic Fuzzy trust approach has the power to manage uncertainty and the ability to present both attributes and results in an easily understandable linguistic form, it was used in this research. 

### 5.4. A Method for Calculating the Value of Merit of eHealth Services

**Figure 3:**
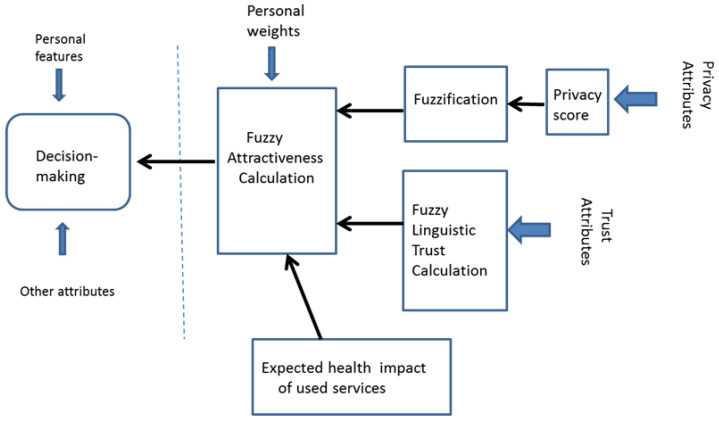
Calculation of the Merit of eHealth service.

The solution developed by the authors can be used by the SerU to calculate a contextual value of Merit (Fuzzy attractiveness rating, FAR) for a selected health service and other participating stakeholders (Figure 3). The SerU can use the calculated value of Merit in the decision to use or not to use the service. 

In this research, the computational Fuzzy linguistic method, developed by Lin at al., was used for FAR calculation, and the formative measurement approach for the selection of attributes was applied in the calculation [61,82]. FAR was calculated using Equation (1) [56]. In the calculation, three variables were deployed: the service computational privacy score, trust rating, and expected health impact of service (EXPHI). The SerU’s psychological and personal factors and impacts of marketing were not included because these are difficult or impossible to measure. To simplify the calculation, the Fuzzy triangular membership function was used. The privacy score was calculated as the numeric (crispy) average of selected attributes, and it was transformed into a linguistic Fuzzy number using a method proposed by Delgado et al. [83,84,85]. The Fuzzy trust number is a simple Fuzzy average of the linguistic values of the trust attributes.

(1) $$\mathrm{FAR}=\sum_{j=1}^{n}(\mathrm{Wj}\;⊗\;\mathrm{Rj})/\sum_{j=1\;}^{n}\mathrm{Wj}$$

where W_j_ is the personal weight for j’s attribute and R_j_ is the Fuzzy linguistic rating for j’s attribute.

The calculated FAR was itself a Fuzzy number. To make its meaning easily understandable for a human it was matched to the linguistic labels used earlier for trust. Additionally, the label whose meaning was closest to the meaning of the FAR number was selected for the proxy for FAR. Different methods such as the Fuzzy similarity measurement and Fuzzy set distance measurement can be used for this transformation [61,86,87]. As the Euclidian method requires the use of alpha cuts, a mathematically easier method using center-of-gravity points of Fuzzy numbers was deployed in this paper. According to Zhang, the value of full similarity of two Fuzzy sets in this method is “1” [86].

### 5.5. Information Sources and Quantification of Privacy and Trust

The main challenge in FAR calculation is the availability and usability of attributes. Furthermore, the attributes used should be easy to use and to understand for a human, and the number of attributes should be kept low. Furthermore, attributes should be, if possible, directly measurable, matching both SerU’s privacy and trust concerns, and be in line with previously selected trust models (Chapter 5). Based on our performed literature analysis, a summary of available sources for privacy and trust attributes is shown in Table 2. 

The optimal solution is to measure the level of actual privacy. As mentioned earlier, this is nearly impossible for the SerU. Therefore, proxy variables (legal norm responsibilities and privacy policies) are used instead (Chapter 5.3). Their attributes can be extracted from available sources such as policy documents, certificates and audit documents (Table 2). Third party seals and the use of data encryption in communication can be also exploited. The literature analysis performed by the authors resulted in a summary of eHealth user’s privacy needs and how they are expressed in privacy policy documents and privacy law (Appendix A).

Researchers have intensively studied the privacy policy documents of organizations. Wilson et al. found that privacy policies vary in length, complexity, legal sophistication, and coverage of services, and the majority of them are unstructured, making their analysis difficult for a human [98]. In real life, privacy policies are usually long narrative documents written in legalese [99]. According to Pollach, the primary goal of policy documents is to protect companies against privacy lawsuits [100]. Iwaya et al. noted that policy documents are commonly publicly available on the service provider’s website, and a level of communication privacy can be estimated from their content [101]. Oltramari et al. note that privacy policies are legally binding documents [102]. Researchers have found that the main challenge for the content analysis of policy documents is to select a useful granularity. According to Harkous et al., researchers have proposed the use of 10 classes for privacy policy analysis; however, in his Polisis solution, 122 privacy classes were used. For a human, such a large number of factors can be confusing. However, computer-based automatic analysis seems to be a promising solution [103].

Based on the performed literature analysis and previous discussions, the authors state that policy document analysis is a suitable tool to identify privacy attributes. In this research work, privacy attributes for eHealth services were selected using heuristic analysis. The findings are shown in Appendix A, and the proposals made by Egger, Oltamari, Harkous, Costance, and Beke [102,103,104,105] were used to select the privacy attributes applied (Table 3).

Encryption is applied as proxy for communication privacy, and audit trails as proxy for transparency. To support the privacy as property approach discussed earlier, the SerU can present their own privacy needs (i.e., how PHI should be processed) by selecting one of three possible values shown in Table 4.

Researchers have proposed a huge amount of trust attributes for e-commerce, Internet shopping and online services, such as personality-based, sociological, provider-specific, technology- and IT-system-specific, institutional, structural, information, service type and quality-based features. Pennanen presented 49 different antecedents in 3 categories: interpersonal (22); institutional (8); and consumer-specific (19) [38]. Hussin et al. classified trust attributes in 7 groups: information-based (25 attributes); function-based (6); merchant-based (15); content-based (4); product-based (4); process-based (4); and others (36). He mentioned that a company’s information, such as address, e-mail address, privacy policy, third-party seals for secure transactions for personal data protection, and third-party recommendations were the most important factors [106]. For organizations, Söllner found 53 attributes: 6 for institutions and 11 for IT [107]. Beldad et al. classified trust attributes in online services into 10 categories (e.g., customer-/client-based, website-based, and company-/organization-based) [39]. Rocs et al. found that perceived ease of use, perceived usefulness, and perceived security are important determinants for trust in online systems [108]. In a review made by Arifim et al., 34 trust antecedents were identified. Most commonly cited were expertise, reputation, experience, frequency of interactions, confidential communication, similarity, integrity, dependability, length of relationship, and firm size [109]. Tamini et al. found that factors such as reliability, assurance, credibility, product type, experience, reputation, personality type, and cultural background were main drivers for e-trust [110]. In a literature analysis, Ruotsalainen et al. found 58 trust attributes classified into the following groups: customer perception and experiences, characteristic of the service provider, service features, and information-based features and infrastructural factors [111]. McKnight el. al. proposed structural assurance and situational normality of an organization as trust attributes [80].

The authors’ literature analysis of eHealth publications found 38 different trust attributes in 5 categories: personal elements and individual antecedents (5); website-related antecedents (9); service provider-related elements (20); informational elements, i.e., design and content factors (9); and information sources (5) (Appendix B). A meaningful finding was that informational elements were the most meaningful attributes in eHealth [112]. According to Liu et al., direct experience is the most reliable information factor for trust measurement [18]. In the case of unavailability of that information, second-hand knowledge and perceptions [113], as well as existing knowledge and evidence [114], can be used. Chen et al. noted that customer’s expectations of a seller’s future behavior are determined by an evaluation of the seller’s past behavior, intentions, capabilities, and values [36]. 

As discussed in Chapter 5.3, a computational trust approach was used in this research. Considered trust attributes included direct measurements, past personal experiences, observed information, transaction ratings, public knowledge (reputation), experts’ recommendations and reports, and users’ perceptions [9,17,88,97,115,116,117]. In real life, perceptions describing the relationship between a trustor and a trustee are widely used as a proxy for trust [43]. A challenge with perceptions is that their sources can remain unclear, and it can be difficult for a person to separate perceptions from beliefs. Furthermore, perceptions do not fully guarantee the service provider’s actual trust features and trust behaviors. In spite of these limitations, according to Li et al., perceptions and second-hand knowledge (e.g., reputation and expert opinions) can be used as proxy for trust in situations where direct and previous information are not available [113]. 

A heuristic method using the content of Appendix B and the findings discussed above were deployed in the selection of five trust attributes for FAR calculation (Table 4). 

The third variable used in FAR calculation, i.e., the expected health impact of services (EXPHI), can be understood as an estimate of expected quality of service (QoS).

### 5.6. Case Study

**Figure 4:**
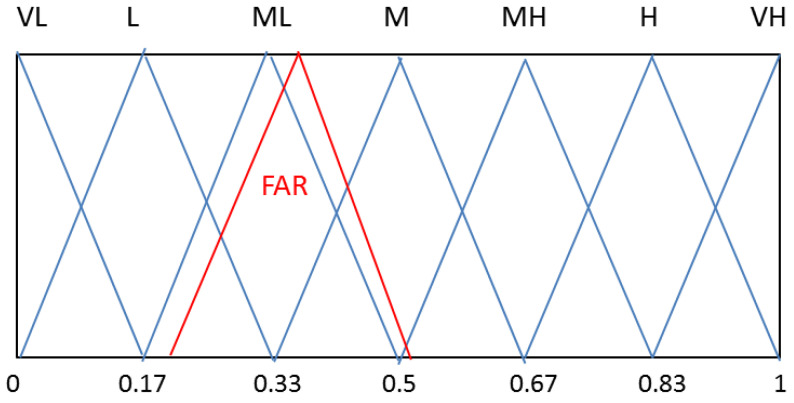
Used membership function and labels.

In a case study, a SerU found an interesting website of a health service that seemed to offer personal health benefits. For the calculation of trust and EXPHI, the following linguistic labels and triangular membership functions (set S) were used (Figure 4). 

For the Set S, the following values were selected: Very low (VL) (0, 0, 0.17); Low (L) (0, 0.17, 0.33); Lower than average (ML) (0.7, 0.33, 0.5); Average (M)(033, 0.5, 0.67); Higher than average (MH) (0.5, 0.67, 0.83); High (H) (0.67, 0.83, 1); Very High (VH) (0.83, 1, 1). For personal weights (W) for privacy, trust and EXPHI, the following labels were selected: Very Low (VL) (0, 0, 0.4); Low (L) (0, 0.4, 0.6); Average (M) (0.4, 0.6, 0.8); High (0.6, 0.8, 1); and Very High (VH) (0.8, 1, 1).

In this case, the user selected the following privacy (“P”) and trust (“T”) ratings for the eHealth website studied (P_i_ is i privacy rating and T_j_ is j trust value). Furthermore, the linguistic value “M” was selected for the expected health impact (EXPHI) (Table 5).

The average of the privacy attributes had the value 0.15. This crisp number was transformed into a Fuzzy number using the method presented by Herrera et al. [79]. The two tuples that represent the information of 0.15 are shown in set S→(L, −12). This indicates that linguistic level L (Low) in set S is an acceptable approach for the number 0.15. In this use case, the user selected the following linguistic weights: privacy = VH; Trust = H; and EXPHI = M. The calculated Fuzzy numbers and their corresponding weights used in the FAR calculation are shown in Table 6. Using Equation (1) for FAR calculation (Chapter 5.4.1), the Fuzzy value for FAR was (0.198, 0.376, 0.56) (Table 6).

To present FAR in set S, a similarity calculation using the center-of-gravity method (i.e., similarity of two Fuzzy numbers) was performed [86]. It produced the following similarities: S_COG_ (FAR, L) = 0.70, S_COG_ (FAR, ML) = 0.92 and S_COG_ (FAR,M) = 0.77. Therefore, the Fuzzy label “ML” is a good linguistic estimate for the Merit of Service (see Figure 4).

## 6. Discussions

Millions of people use the Internet and mobile eHealth services and applications. Furthermore, an increasing number of regulated healthcare organizations are moving part of their services to digital networks and ecosystems. To be effective, these services require the availability of an extensive amount of PHI. These, and situations where eHealth services are part of an ecosystem, raise many security and trust concerns. The disclosure of sensitive PHI requires that the SerU knows in advance the level of privacy in the ecosystem, and why and how much she or he can trust the SerP and the other stakeholders in the ecosystem. Trust requires data about the other partners [118] and knowledge of the ecosystem’s privacy features. In real life, it is difficult for the SerU to know the actual level of privacy and trust offered by the ecosystem, and to make informed decisions. There is often a lack of reliable and directly measurable privacy and trust information. In this situation, humans are subject to psychological deviations from rationality, and individuals often mispredict their own preferences, derive inaccurate conclusions, or make inappropriate decisions [119].

To help SerUs in making information-based decisions regarding whether or not to use eHealth services, the authors developed a solution that calculates the Merit of Service value for the eHealth service and the surrounding ecosystem. The solution uses available information and perceptions concerning the SerP’s and ecosystem’s privacy and trust features and behaviors. For calculation, a Fuzzy linguistic method that used available or measurable attributes was deployed. Privacy attributes were derived from the service provider’s privacy policy documents, and trust was estimated from the available trust-related information and from user’s trust perceptions. Personal weights were also supported. The solution was user friendly, as linguistic labels were applied for trust attributes and for the value of Merit. The solution was automated, i.e., it can be given by a computer application that autonomously collects most/all data needed for the calculation. The solution was also flexible, so different privacy and trust models and context-specific attributes can be used. The service user can use the FAR value as an input to the final decision-making process to use or not to use the offered eHealth service. In this way, the FAR is—from the service user’s point of view—a step forward from the current unsatisfactory situation. Considering the possible dissemination of the developed method, the next step might be the development of an open-source application, made freely available for testing in real-life situations.

The solution has also weaknesses. Caused by the lack of reliable information of actual privacy and trust, proxies were used. The availability of the service provider’s privacy documents and trust promises does not fully guarantee that the provider keeps their promises. Furthermore, privacy documents are often high-level documents which do not explain the level of situational normality (i.e., which privacy safeguards are in place). E-commerce research has shown that a user’s trust in service providers can be manipulated in many ways. For example, the appearance of a website impacts a user’s trust, and the recommendations of others can be manipulated [120]. A weakness of the current solution is also that, currently, a SerU has to analyze the SerP’s privacy documents manually, which can be time consuming, difficult and frustrating. Policy analysis using artificial intelligence (AI) and machine learning is a promising solution to this problem [102,103,104].

Two remaining barriers to this solution are: firstly, the lack of reliable and accurate privacy and trust information available; secondly, regulators’ low willingness to force service providers and other stakeholders of the ecosystem to make reliable and detailed information concerning their privacy and trust features freely available. This unsatisfactory situation will continue as long as service providers do not have incentives to publish this information to enable the measurement of actual levels of privacy and trust. 

The question as to whether there are risks when using FAR values (i.e., the possibility that physical, social or economic harm can be caused) also needs attention. The FAR value is generated by a computational algorithm that can be voluntarily used in decision-making. It differs from machine learning algorithms because, in the FAR method, the user defines personal weights. Based on these features, the authors consider it unlikely to cause harm to the service user.

The authors’ solution is a step towards the trustworthy and privacy-enabled use of eHealth services. It highlights the development of new intelligent tools for the SerU in managing information privacy and creating trust in eHealth and in other digital services offered in ecosystems. Political will is needed to change the current regime that enables the collection and use of PHI against a user’s personal preferences and privacy laws [11].

## Figures and Tables

**Table 1:** Specific features of eHealth ecosystems.

Highly sensitive health-related data (e.g., diseases, symptoms, social behavior, and psychological features) are collected, used and shared
Healthcare-specific laws regulate the collection, use, retention and disclosure of PHI
To use services, the user must disclose sensitive PHI
Misuse of PHI can cause serious discrimination and harm
Service provided is often information, knowledge or recommendations without quality guarantee or return policy
The service provider can be a regulated or non-regulated healthcare service provider, wellness-service provider or a computer application
Service user can be a patient, and there exists a fiducial patient–doctor relationship

**Table 2:** Typical sources for privacy and trust attributes from [7,54,88,89,90,91,92,93,94,95,96,97].

Direct measurements, experiences, interactions and observations
Service provider’s privacy policy document
Content of privacy certificate or seal for the medical quality of information, content of certificate for legal compliance (structural assurance), andaudit trial (transparency).
Past experiences, transaction history, previous expertise
Information available on service provider’s website
Provider’s promises and manifestations
Others recommendations and ratings, expected quality of services
Information of service provider’s properties and information system
Vendor’s type or profile (similarity information)

**Table 3:** Selected privacy attributes and their possible values.

Name	Meaning of Attribute	Value = 2	Value = 1	Value = 0
P1	PHI disclosed to third parties	No data disclosed to third parties	Only anonymous datais disclosed	Yes/no information
P2	Regulatory Compliance	Compliance certified by experts third-party privacy seals	Demonstrated regulatory complianceAvailable	Manifesto or no information
P3	PHI Retention	Kept no longer than necessary for purposes of collection	Stored in encrypted form for further use	No retention time expressed
P4	Use of PHI	Used only for presented purposes	Used for other named purposes	Purposes defined by the vendor
P5	User access to collected PHI	Direct access via network	Vendor made document of collected PHI is available on request	No access or no information available
P6	Transparency	Customer has access to audit trail	No user access to audit trail	No audit trail or no information
P7	Ownership of the PHI	PHI belongs to DS (user)	Shared ownership of PHI	Ownership of PHI remains at vendor or no information
P8	Support of SerU’s privacy needs	SerU’s own privacy policy supported	Informed consent supported	No support of DS’ privacy policies or no information
P9	Presence of organisation	Name, registered office address, e-mail address and contact address of privacy officer available	Name, physical address, e-mail address available	Only name and e-mail address available
P10	Communication privacy	End-to-end encryption for collected PHI	HTTPS is supported	Raw data collected or no information

**Table 4:** Selected trust attributes for FAR calculation.

Name	Attribute	Meaning	Sources
T1	Perceived Credibility	How SerP keeps promises, type of organisation, external seals, ownership of organisation	Previous experiences, website information
T2	Reputation	General attitude of society	Websites, other sources
T3	Perceived competence and professionalism of the service provider	Type of organisation, qualification of employees/experts, similarity with other organisations	Website information, external information
T4	Perceived quality and professionalism of health information	General information quality and level of professionalism, quality of links and scientific references	Own experience, third party ratings, other’s proposals, website information,
T5	Past experiences	Overall quality of past experiences	Personal past experiences
T6	Regulatory compliance	Type and ownership of organisation. Experiences how the SerP keeps its promises	Websites, oral information, social networks and media. Previous experiences
T7	Website functionality and ease of use	Easy to use, usability, understandability, look of the website, functionality	Direct experiences
T8	Perceived quality of the information system	Functionality, helpfulness, structural assurance, reliability (system operates properly)	Own experiences, others recommendations

**Table 5:** Privacy and trust ratings and EXPHI value example.

P1 = 0.	P2 = 0	P3 = 0	P4 = 1	P5 = 0	P6 = 0	P7 = 0	P8 = 0	P9 = 1	P10 = 1
T1 = M	T2 = MH	T3 = ML	T4 = M	T5 = H	T6 = L	T7 = H	T8 = M		EXPHI = M

**Table 6:** Linguistic values for calculation of FAR.

Factor	Fuzzy Value	Fuzzy Weight
Privacy	L (0.0, 0.17, 0.33)	VH (0.8, 1, 1)
Trust	(0.375, 0.54, 0.71)	H (0.6, 0.8, 1)
EXPHI	M (0.33, 0.5, 0.67)	M (0.4, 0.6, 0.8)
**FAR**	**(0.198, 0.376, 0.562)**	

## Data Availability

Not applicable.

## Appendix A

**Table A1:** Privacy Needs and Requirements In Policy Documents and Law from [95,102,103,104,105,121].

Privacy Needs/Questions	Meaning in a Privacy Policy Document	Requirements Exressed by Law (General Data Protection Regulation, EU GDPR) ^1^
PHI used only for purposes defined by the service provider	How and why a service provider collects and uses PHI	Limited by what is necessary in relation to purpose. Explicit purpose
PHI not disclosed to third parties	What data and how PHI is shared with third party	Personal policiesTransparency
Regulatory compliance	Level Regulatory compliance	Lawfully processing Demonstrate regulatory compliance
What is the content of a personal privacy policy?	Edit and deletion	Erase, right to become forgotten, right to object processing, explicit purpose
What are the service provider’s characteristics?	Type of organisation address	
Encryption	Communication privacy	Encryption
How PHI is stored for future use	Data retention (stored as long as needed to perform the requested service/indefinitely)	Retention no longer than necessary for purpose
User access to audit trail	What data is shared/transparency	Lawfully processing and transparency
User access to own PHI	User access, rights to view records	Access to collected PHI. Right to erase and object processing
How personal privacy needs are supported	User choice/control (consent, Opt in/opt out, purpose)	Accept personal privacy policies/explicit consent
Does PHI belongs to the customer?	Ownership of data	The individual owns the rights to their data
Does a registered office and address exist?	Contact information	
Privacy guarantees	Third-party seals or certificates	
Transparency	Transparency	Right to become informed

^1^ The General Data Protection Regulation (GDPR) is an EU-wide privacy and security law put into effect on 25 May 2018.

## Appendix B. Trust Attributes for eHealth

**Personal elements and individual antecedents** from [32,49,112,122]

- General trust of the health website;
- Personality;
- Privacy concerns;
- Subjective belief of suffering a loss;
- Beliefs in ability, integrity and benevolence.

**Website-related antecedents** from [32,49,112,122,123,124]

- Website design and presence, website design for easy access and enjoyment;
- System usability, perceived as easy to use;
- Technical functionality;
- Website quality (being able to fulfil the seekers’ needs);
- Perceived information quality and usefulness;
- Quality (familiarity) that allows better understanding;
- Simple language used;
- Professional appearance of the health website;
- Integrity of the health portal policies with respect to privacy, security, editorial, and advertising.

**Service provider (institution, non-profit organisation, private business)-related elements** from [32,49,112,122,123,124,125,126]

- Credibility and impartiality;
- Reputation;
- Ability to perform promises made;
- Accountability of misuse;
- Familiarity;
- Branding, brand name and ownership;
- System quality (functionality flexibility), quality of systems, stability;
- Professional expertise;
- Similarity with other systems, ability, benevolence, integrity of the health portal with the same brand;
- Transparency, oversight;
- Privacy, security;
- Privacy and security policies, strategies implemented;
- Regulatory compliance.

**Informational elements (design and content factors)** from [49,112,122,124]

- Quality of links;
- Information quality and content (accuracy of content, completeness, relevance, understandable, professional, unbiased, reliable, adequacy and up-to-date), source expertise, scientific references;
- Information source credibility, relevant and good information, usefulness, accuracy, professional appearance of a health website;
- Information credibility;
- Information impartiality.

**Information sources** from [49,67,112,122,127]

- Personal interactions;
- Personal experiences;
- Past (prior) experiences;
- Presence of third-party seals (e.g., HONcode, Doctor Trusted™, TrustE).
